# Effect of liraglutide on the dysglycemia, inflammation, and gut microbiota in prediabetic KKay mice

**DOI:** 10.3389/fphar.2025.1714859

**Published:** 2025-11-28

**Authors:** Ying Zhang, Xiaoxiao Yang, Ping Yang, Huihuan Sun, Lijuan Chen, Xiaojun Zhang, Shudong Liu

**Affiliations:** 1 Department of Endocrinology, Shandong Rongjun General Hospital, Jinan, China; 2 Department of Cardiology, Shandong Rongjun General Hospital, Jinan, China

**Keywords:** prediabetes, dysglycemia, inflammation, GLP-1RA, gut microbiota, bioinformatic analysis

## Abstract

Prediabetes is a significant risk factor for type 2 diabetes mellitus (T2DM). Emerging evidence suggests that glucagon-like peptide-1 receptor agonists (GLP-1RAs) may modulate the gut microbiota and improve dysglycemia in T2DM. In this study, we investigated the effects of liraglutide on dysglycemia and gut microbiota in prediabetic mice. KKay mice were fed a high-fat diet to establish prediabetes. The prediabetic mice were then treated with a daily intraperitoneal injection of liraglutide for 12 weeks. 16S rDNA sequencing was employed to investigate alterations in the gut microbiota in prediabetic mice and liraglutide-treated prediabetic mice. The gut bacterial metabolites in the ileal contents of prediabetic mice were measured via a liquid chromatography‒mass spectrometry (LC‒MS) system. Prediabetic mice presented significantly increased body weights, blood glucose levels, and inflammatory factor levels and decreased GLP-1 levels. Liraglutide treatment improved dysglycemia and insulin secretion and inhibited systematic and tissue inflammation in prediabetic mice. Prediabetic mice presented pronounced increases in the abundance of *f_Ruminococcaceae*, *g_Anaerotruncus*, *s_Anaerotruncus_sp_G3_2012, s_Ligilactobacillus_murinus*, *s_Desulfovibrio_fairfieldensis, g_Ligilactobacillus*, *g_Parabacteroides*, *g_Butyricimonas*, and *g_unclassified_Ruminococcaceae*. Liraglutide treatment changed the intestinal microbiota composition and related signaling pathways. Our preliminary results demonstrate that GLP-1RA liraglutide exerts beneficial effects by improving dysglycemia and body weight, inhibiting inflammation, and modulating gut microbiota in prediabetic mice, potentially contributing to delaying or preventing the progression from prediabetes to overt diabetes.

## Introduction

Prediabetes is an intermediate state of dysglycemia that lies between euglycemia and diabetes (Echouffo-Tcheugui et al., 2023). Prediabetes is also a significant risk factor for cardiovascular disease, type 2 diabetes mellitus (T2DM), tumors, and dementia (Salinero et al., 2020; Echouffo-Tcheugui et al., 2023; Yu and Wan, 2024). Surveys conducted in China have indicated that in 2013, the prevalence of diabetes among adults was 10.9%, whereas the prevalence of prediabetes was 35.7% (Wang et al., 2017). Five to ten percent of individuals with prediabetes are expected to progress to diabetes each year, leading to as many as 70% of those with prediabetes ultimately developing diabetes (Tabak et al., 2012). Prediabetes is reversible, and effective early intervention in individuals with prediabetes can reduce the risk of developing T2DM by 40%–70% (Tabak et al., 2012). Hence, early intervention and management of prediabetes is highly important for the prevention and control of diabetes.

Glucagon-like peptide-1 (GLP-1) is a peptide hormone secreted by neuroendocrine L cells located in the terminal ileum, colon, and rectum (Andersen et al., 2018). A growing body of clinical evidence has demonstrated that reduced GLP-1 secretion is significantly associated with the development of T2DM and prediabetes (Faerch et al., 2015). Currently, GLP-1 receptor agonist (GLP-1RAs), such as liraglutide, are being used clinically and can effectively reduce fasting blood glucose levels, postprandial blood glucose levels, weight, and glycated hemoglobin (HbA1c) levels in T2DM patients (Yao et al., 2024). GLP-1RAs can increase insulin secretion, lose weight, and promote satiety (Kim et al., 2024; Wong et al., 2024; Duan et al., 2024). Recent studies have revealed that GLP-1RAs may also exert significant effects in prediabetic patients (Perreault et al., 2022; Le Roux et al., 2017). However, the molecular underpinnings of the actions of GLP-1RAs in prediabetes patients are not fully understood.

Recently, several clinical studies have revealed that dysbiosis of the gut microbiota is closely associated with development of prediabetes and T2DM (Wu et al., 2020; Takeuchi et al., 2023; Chang et al., 2024; Gravdal et al., 2023). The gut microbiota and its metabolites are involved in the regulation of insulin resistance and insulin sensitivity through multiple mechanisms, including inflammatory factors, microbial metabolite-related networks, and the immune response (Takeuchi et al., 2023). Modulation of gut microbiota could improve insulin sensitivity, hyperglycemia, and lipid metabolism (Qi et al., 2025). Studies have shown that probiotics and metabolites derived from gut bacteria, short-chain fatty acids (SCFAs) such as acetate and butyrate, bile acids, and indole, can inhibit the formation of inflammatory cytokines and stimulate the secretion of GLP-1 (Yan et al., 2025; Xia et al., 2024; Zhang et al., 2023). In addition, GLP-1RA liraglutide represents an effect on the gut microbiota composition and promotes bacterial translocation in dysmetabolic and obese mice (Kato et al., 2021; Charpentier et al., 2021; Moreira et al., 2018). However, the interaction between GLP-1RA and gut microbiota in prediabetes remains unclear.

Therefore, the main aim of the present study was to investigate how the composition of the gut microbiota changes in prediabetic mice and to investigate the effects of liraglutide on gut microbiota profiles and metabolites. Exploring the underlying mechanism of GLP-1RA in prediabetes is helpful for providing a deeper theoretical basis for early intervention and treatment in prediabetic patients.

## Materials and methods

### Mouse experiments

In the study, four-week-old specific pathogen-free (SPF)-grade KKay male mice were purchased from Xinbainuo Biotechnology Co., Ltd. (Jinan, China), and age-matched male C57BL/6J mice were obtained from Charles River Laboratories (Beijing, China). The animal experiments were performed in accordance with the animal welfare guidelines and the Animal Research: Reporting of *In Vivo* Experiments (ARRIVE) guidelines. All animal experimental procedures were approved by the Ethics Committee of Shandong Rongjun General Hospital (No: RY00006).

The mice were maintained in an SPF room with *ad libitum* access to water on a 12-h light/dark cycle. All animals are housed five per cage and labeled with coded identifiers (ear tags). After acclimatization for 1 week at 5 weeks of age, KKay mice were placed on a high-fat diet (HFD, 60% energy from fat, 20% protein, 20% carbohydrate; Research Diet: D12492) for 3 weeks to induce a prediabetic state (Liu et al., 2020). A Roche glucometer was used to measure weekly blood glucose levels in 12-h (h)-fasted mice via the tail vein. Mice with fasting glucose level less than 11.1 mmol/L were considered to meet the standard of prediabetes for the next step of the study. KKay mice with blood glucose levels above 11.1 mmol/L were excluded from the present study (Zborowski et al., 2021; Ren et al., 2023; Zhang et al., 2024b).

Subsequently, twenty prediabetic KKay mice were enrolled and randomly divided into two groups using a computer-generated randomization schedule (n = 10 per group). The two groups were as follows: (1) the prediabetes model group was fed an HFD for 12 weeks; (2) the prediabetes + liraglutide (Lira) group was fed an HFD and treated with a daily intraperitoneal injection of liraglutide (Victoza; Novo Nordisk, Bagsvaerd, Denmark) at a dose of 200 μg/kg for 12 weeks (Zhao et al., 2024). Ten control C57BL/6J mice were fed a normal diet until the end of the experiment. During the 12 weeks of treatment, body weights and fasting blood glucose levels were measured every 2 weeks. The homeostasis model assessment of insulin resistance (HOMA-IR) index was assessed insulin resistance based on the following formulas: fasting glucose (mmol/L) × fasting insulin (µU/mL)/22.5 (Liao et al., 2019).

At the end of the treatment period, retroorbital blood samples were collected after an overnight (12 h) fast. For active GLP-1 analysis, blood samples were collected into prechilled tubes containing K2- ethylenediaminetetraacetic acid (EDTA) plus sitagliptin (freshly prepared; 50 μmol/L), an anti-dipeptidyl-peptidase-IV inhibitor, which could inhibit GLP-1 degradation (Millipore, Billerica, MA, United States) as previously described (Wang et al., 2018). Then, the mice were sacrificed by cervical dislocation. The ileal contents were immediately collected and frozen in liquid nitrogen, and transported to Sibei Biotechnology (Shandong) Co., Ltd. on dry ice for microbial DNA extraction and analysis (n = 5 per group). Ileal tissue samples were immediately frozen in liquid nitrogen and stored at −80 °C for subsequent analyses (n = 10 per group).

### Intestinal microbiota analysis

Total genomic DNA was extracted from ileal content samples (n = 5 per group) using a TGuide S96 Magnetic Soil/Stool DNA Kit (Tiangen Biotech (Beijing) Co., Ltd.) according to the manufacturer’s instructions. 2-chlorophenylalanine (0.2 mg/mL) was as an internal standard. The quality and quantity of the extracted DNA were analyzed via 1.8% agarose gel electrophoresis, and the DNA concentration and purity were determined using a NanoDrop 2000 UV‒Vis spectrophotometer (Thermo Scientific, Wilmington, United States). The hypervariable V3-V4 region of the bacterial 16S rRNA gene was amplified using the primer pair 338F: 5′- ACT​CCT​ACG​GGA​GGC​AGC​A-3′ and 806R: 5′- GGACTACHVGGGTWTCTAAT-3′. The polymerase chain reaction (PCR) products were subjected to agarose gel electrophoresis and purified using an Omega DNA purification kit (Omega Inc., Norcross, GA, United States). The purified PCR products were collected, and paired-end sequencing (2 × 250 bp) was performed on an Illumina NovaSeq 6000 platform at Biomarker Technologies Co., Ltd. (Beijing, China) (Modi et al., 2021).

### Raw data processing and bioinformatic analysis

Raw data processing included the following 2 steps. (1) Raw read filtration: Raw reads were first filtered by Trimmomatic v 0.33, and then the primer sequences were identified and removed by cutadapt 1.9.1, which finally generated high-quality reads without primer sequences; (2) DADA2 denoising: Data was processed using dada2 (Callahan et al., 2016) in the R library to denoise and remove chimeric sequences, generating nonchimeric reads. Bioinformatic analysis includes feature.

### Identification (amplicon sequence variants (ASVs)), diversity analysis, differential analysis, correlation analysis and functional prediction

The qualified sequences with more than 97% similarity thresholds were allocated using dada2 to generate ASVs, and the samples with ASV counts less than 2 were filtered (Callahan et al., 2016). Taxonomic annotation of the ASVs was performed via the Naive Bayes classifier in QIIME2 (Bolyen et al., 2019) using the SILVA database (release 138.1) (Quast et al., 2013) with a confidence threshold of 70%. Alpha diversity analysis including the Chao1, Shannon, and Simpson indices was performed utilizing QIIME2 software to identify the complexity of the species diversity of each sample. Beta diversity analysis was performed via principal coordinate analysis (PCoA) to assess the species complexity diversity across the samples.

Metastats analysis was conducted to assess the differences in microbial community abundance at the phylum, class, order, family, genus and species levels for intergroup significance analysis. Linear discriminant analysis (LDA) coupled with effect size (LEfSe) was applied to evaluate the differentially abundant taxa (LDA effective size >4.0 and P < 0.05 are displayed). Kyoto Encyclopedia of Genes and Genomes (KEGG)-based functional prediction was performed using Phylogenetic Investigation of Communities by Reconstruction of Unobserved States (PICRUSt2) software (Kanehisa and Goto, 2000). The online BMKCloud platform (https://www.biocloud.net) was used to analyze the sequencing data.

### Western blotting

Western blotting was performed as previously described (Guo et al., 2023). Briefly, 30 mg of ileal tissue was homogenized and fully lysed in lysis buffer containing protease inhibitors. The protein concentration was determined using a bicinchoninic acid (BCA) assay. Approximately 50 μg protein from each sample was separated by electrophoresis using 10% SDS‒PAGE and then electrotransferred onto polyvinylidene fluoride (PVDF) membranes. The membranes were blocked with 5% nonfat milk in tris-buffered saline with tween-20 (TBST) buffer for 1 h at room temperature and then incubated with a primary anti-GLP-1 antibody (sc-80603, Santa Cruz Biotechnology) at 4 °C overnight. β-actin was used as an internal reference. The membranes were then washed with TBST 3 times and incubated with a horseradish peroxidase-conjugated secondary antibody for 2 h at room temperature. The blots were then detected by chemiluminescence on a C300 analyzer (Azure Biosystems, Dublin, CA, United States).

### Enzyme-linked immunosorbent assays (ELISAs)

ELISAs were performed to assess ileal and fasting serum levels of insulin, C-peptide, interleukin-6 (IL-6), IL-1β, lipopolysaccharide (LPS), nuclear factor κB (NF-κB), tumour necrosis factor α (TNF-α, Cusabio, Wuhan, China), total GLP-1, and active GLP-1 (7–36,7–37) (n = 10 per group). For tissue, the 100 mg ileum tissue was weighed and homogenized in phosphate buffered solution (PBS) with a glass homogenizer. Then the homogenates were centrifuged for 5 min at 5.000 g, 2 °C–8 °C, and the supernatants were assayed immediately. The optical density was determined using a microplate reader set to 450 nm within 5 min. All the procedures were performed according to the manufacturers’ instructions. A total GLP-1 kit was obtained from Nebula Biochemicals (Beijing, China), and an active GLP-1 (7–36,7–37) kit was obtained from Abcam (ab121057). The antibody binds the free N-terminus of GLP-1 (7–37) and GLP1 (7–36) amide and shows <0.2% cross-reactivity with GLP-1 (9–36)amide, cross-reacts 0.25% with GLP1 (1–37) (Odongo et al., 2023).

### Intraperitoneal glucose tolerance test (IPGTT)

The IPGTT was conducted as previously described with modification (Qureshi et al., 2023; Patel et al., 2024). Mice were injected intraperitoneally with glucose (2 g/kg body weight) when the mice were fasted for 10 h (n = 6 per group). Blood samples were obtained from the tail vein at 0, 30, 60, and 120 min for detecting the blood glucose levels using Roche glucometer (Guo et al., 2023). Area under the curve (AUC) of IPTGG was calculated using the GraphPad Prism 9.0 (GraphPad Software, Inc., San Diego, CA, United States).

### Confocal microscopy

Confocal images were obtained on a Nikon Ti confocal laser scanning microscope and an Eclipse C2 imaging system (Nikon, Tokyo, Japan) (Allmon and Esbaugh, 2017). AIpathwell® software (Servicebio Technology Co. Ltd., Wuhan, China) was used to assess the staining intensities (Wang et al., 2023). The primary antibody used was anti-GLP-1 antibody (Servicebio, Wuhan, China), and the nuclei were stained with 4′,6-diamidino-2-phenylindole (DAPI).

### Metabolomics analysis

The metabolomics analysis of ileal contents were conducted via a liquid chromatography‒mass spectrometry (LC-MS) system as described previously (Liu et al., 2023). For metabolomic analysis, a total of 50 mg of ileal contents were mixed with 1,000 μL of methanol and acetonitrile solution (1:1) in a 2 mL centrifuge tube, vortexed 30 s, and subsequently a small amount of steel balls was added to crush with a grinder 10 min. Next, the mixture was sonicated 10 min at 4 °C. Then, the sample was placed in the refrigerator at −20 °C for 60 min, and centrifuged (12,000 rpm for 15 min). Eventually, the 500 μL supernatant was collected and dried. 160 μL of methanol and acetonitrile solution was added to the residues for re-dissolution. After centrifugation at 12,000 rpm for 15 min at 4 °C, the 120 μL of supernatant was obtained for detection. 10 µL of supernatant was collected for quality control.

The LC‒MS system was composed of a Waters Acquity I-Class PLUS ultrahigh-performance liquid chromatography and a Waters Xevo G2-XS QToF high-resolution mass spectrometer (Wang et al., 2016). The column used was purchased from Waters Acquity UPLC HSS T3 column (1.8 µm 2.1*100 mm). The injection volume for both positive and negative modes was 2 μL. The raw data collected using MassLynx V4.2 were processed by Progenesis QI software for peak extraction, peak alignment and other data processing operations on the basis of the Progenesis QI software online METLIN database and self-built library for identification.

After the original peak area information was normalized to the total peak area, a follow-up analysis was performed. Principal component analysis and Spearman correlation analysis were used to judge the repeatability of the samples within groups and the quality control samples. The identified compounds were analyzed for classification and pathway information using the KEGG database. According to the grouping information, the differences were calculated and compared, and a t-test was used to calculate the significance (P value) of each difference. The R language package “ropls” was used to perform orthogonal partial least squares discriminant analysis (OPLS-DA) modeling, and 200 permutation tests were performed to verify the reliability of the model. The variable importance in projection (VIP) value of the model was calculated using multiple cross-validations. The method of combining the multiple differences, the P value and the VIP value of the OPLS-DA model was adopted to screen the differentially abundant metabolites. The screening criteria were a fold change (FC) >1.5, a P value <0.05 and a VIP ≥1. False discovery rate (FDR) threshold was set at 0.05. The differentially abundant metabolites associated with significant KEGG pathway enrichment were calculated via a hypergeometric distribution test.

### Statistical analysis

The data are expressed as the mean ± standard error of the mean (SEM). Variance homogeneity and data normality were tested with a Shapiro-Wilk test before any testing. Non-normal data were analyzed using the Kruskal–Wallis test. Normally distributed data were examined by one-way analysis of variance (Tukey’s test) for multiple comparisons; controlling the FDR using the Benjamini–Hochberg method (FDR <0.05) with GraphPad Prism 9.0 (Benjamini and Hochberg, 1995). P values <0.05 were considered statistically significant.

## Results

### Liraglutide improves dysglycemia in prediabetic mice

**FIGURE 1:**
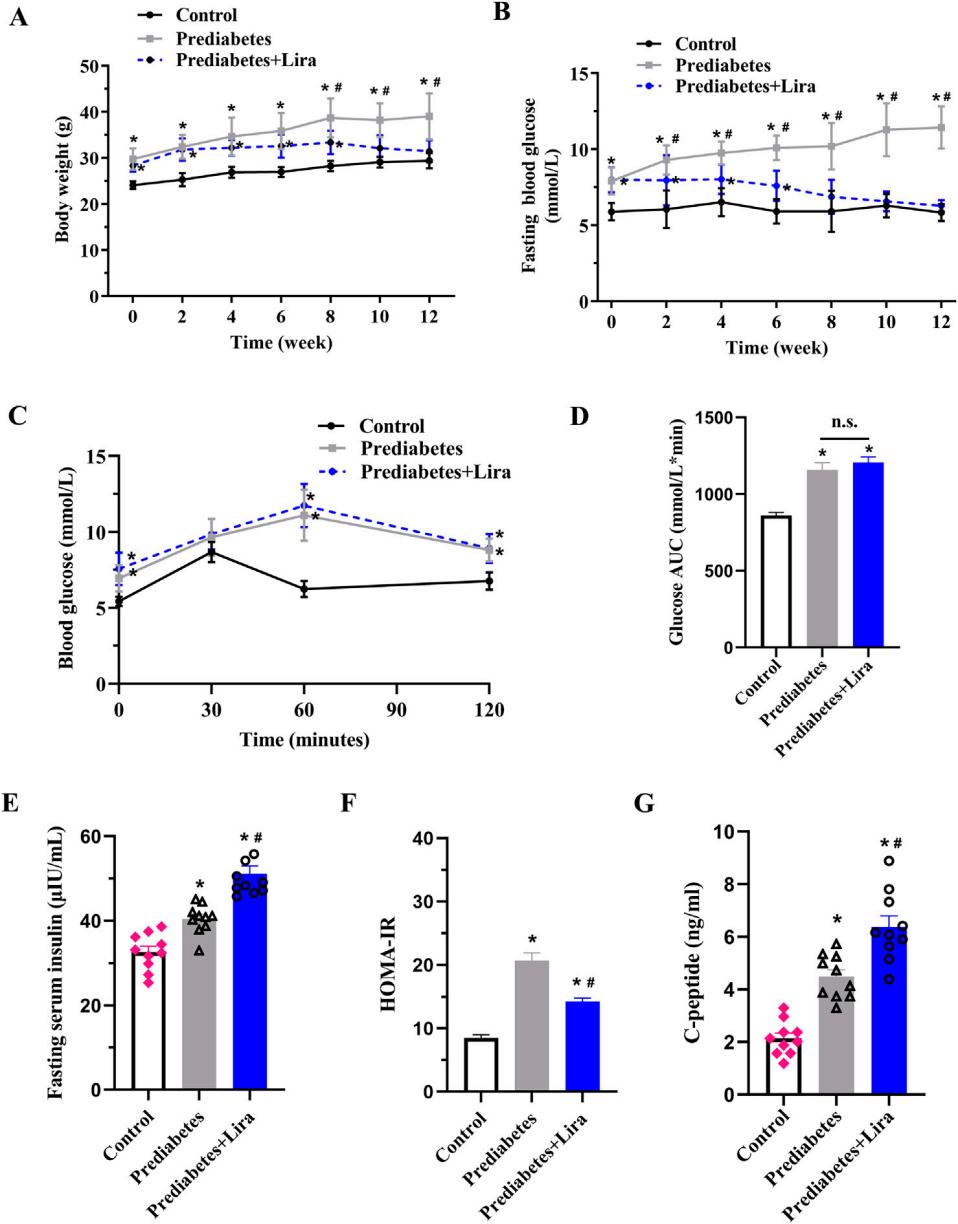
Liraglutide improves dysglycemia in prediabetic mice. Prediabetic mice were fed an HFD followed by 12 weeks of liraglutide (Lira) treatment, and C57BL/6J control mice were fed a normal diet (n = 10 mice per group). **(A)** Body weight. **(B)** Fasting blood glucose. **(C)** IPGTT was conducted at 8 weeks of age in three groups of pre-treatment mice (n = 6 per group). Blood glucose levels were measured at 0, 30, 60, and 120 min. Data are expressed as the mean ± standard deviation (S.D). **(D)** The area under the curve (AUC) for the IPGTT curve. *P < 0.05 vs. control; n.s. denotes no significance. **(E)** Fasting serum insulin levels (n = 10). **(F)** HOMA-IR index (n = 10 per group). **(G)** C-peptide levels (n = 10 per group). The values represent the means ± SEMs. (n = 10 mice/group). *P < 0.05 vs. control, ^#^ *P* < 0.05 vs. prediabetes. n.s. denotes no significance.

To investigate the effect of liraglutide on prediabetes, KKay mice were fed an HFD to induce a prediabetic state and were then subjected to liraglutide treatment for 12 weeks (n = 10 per group). Compared with C57BL/6J control mice, prediabetic mice fed an HFD for 3 weeks presented significantly higher body weights and fasting blood glucose levels (0 weeks) (Figures 1A,B). At 8 weeks of age, IPGTT was conducted. As shown in Figures 1C,D, the blood glucose levels were significantly increased in prediabetic mice before liraglutide treatment. Compared with the control mice and prediabetic mice, liraglutide treatment significantly improved body weights and glucose levels in prediabetic mice at the 10th and 8th weeks, respectively (Figures 1A,B). Additionally, compared with control mice, HFD-fed prediabetic mice presented increased fasting serum insulin, higher HOMA-IR index, and C-peptide levels, while liraglutide treatment significantly increased insulin and C-peptide levels, reduced HOMA-IR index compared with prediabetic mice (Figures 1E–G),suggesting a beneficial effect of liraglutide on glucose homeostasis.

### The effects of liraglutide treatment on ileal and serum inflammatory factors in prediabetic mice

**FIGURE 2:**
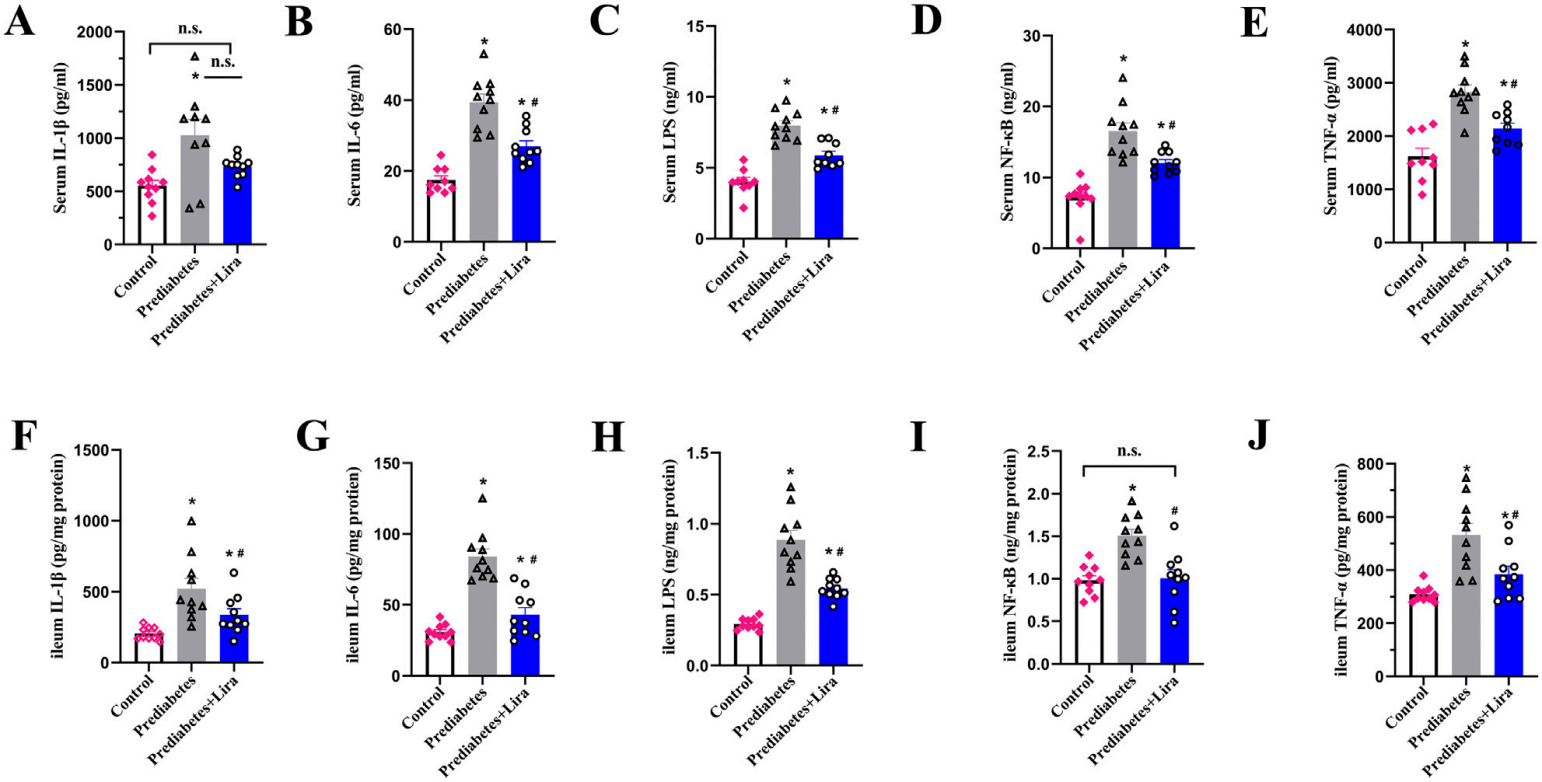
The effects of liraglutide on ileal and serum inflammatory factors. Prediabetic mice were fed an HFD followed by 12 weeks of liraglutide treatment. C57BL/6J control mice were fed a normal diet. **(A)** Serum IL-1β levels. **(B)** Serum IL-6 levels. **(C)** Serum LPS levels. **(D)** Serum NF-κB levels. **(E)** Serum TNF-α levels. **(F)** Ileal IL-1β levels. **(G)** Ileal IL-6 levels. **(H)** Ileal LPS levels. **(I)** Ileal NF-κB levels. **(J)** Ileal TNF-α levels. The values represent the means ± SEMs. n = 10 per group. *P < 0.05 vs. control, ^#^ *P* < 0.05 vs. prediabetes. n.s. denotes no significance.

Previous studies have demonstrated that GLP-1RAs exert anti-inflammatory effects in mice and humans with inflammatory and metabolic diseases (Wong et al., 2022; Charpentier et al., 2021; Alharbi, 2024). We observed that prediabetic mice presented increased levels of inflammatory factors, including IL-1β, IL-6, LPS, NF-κB, and TNF-α, in both the circulation and ileum, and these effects were inhibited by liraglutide treatment (Figures 2A–J); however, there was no significant difference in the serum IL-1β concentration between the prediabetes and prediabetes + Lira groups (n = 10 per group). These results indicate that liraglutide also have beneficial effects on prediabetic mice through the inhibition of tissue and systemic inflammation.

### The effect of liraglutide on GLP-1 secretion in prediabetic mice

**FIGURE 3:**
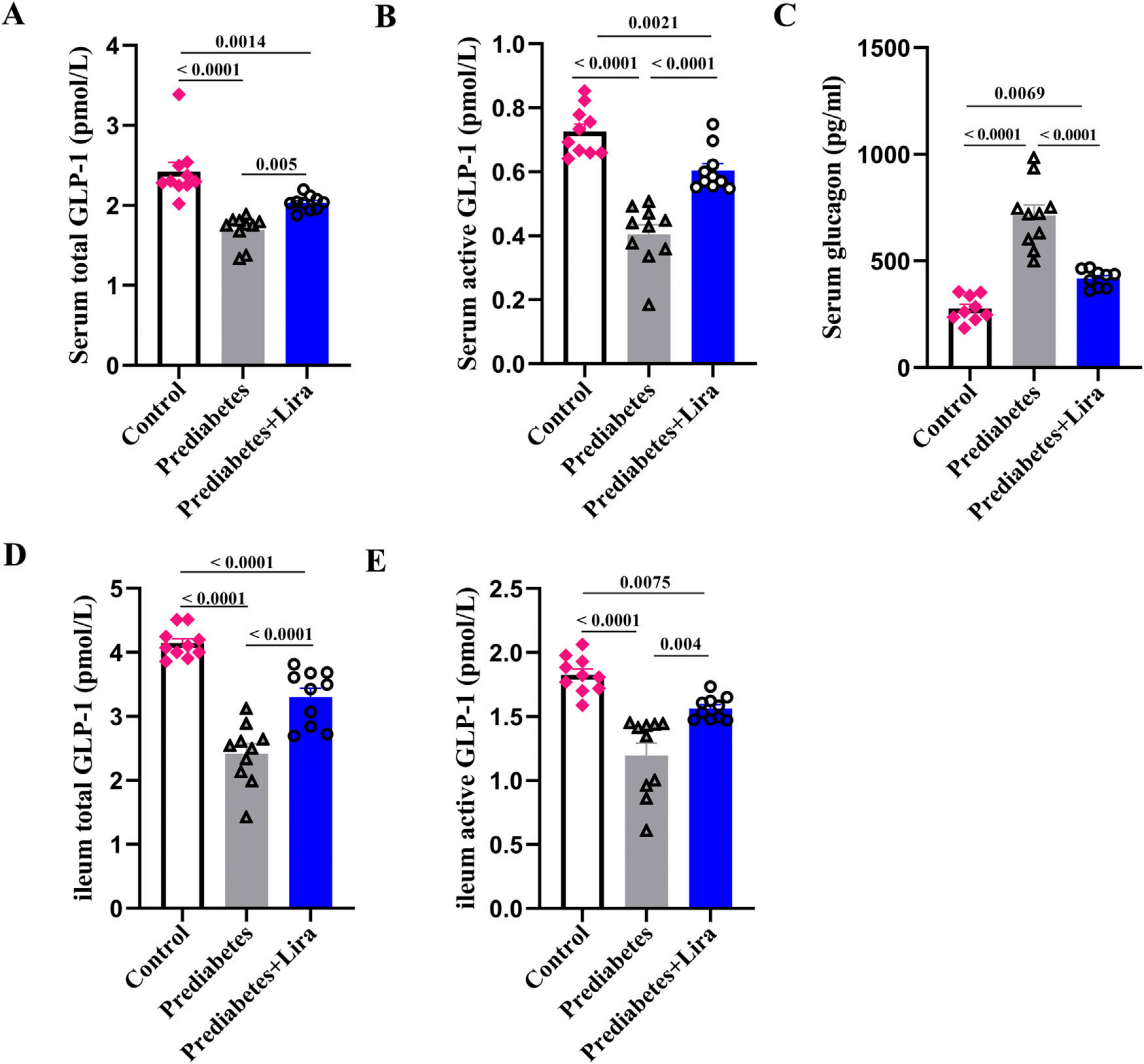
The effect of liraglutide on GLP-1 secretion in prediabetic mice. **(A)** Serum total GLP-1 levels. **(B)** Serum active GLP-1 levels. **(C)** Serum glucagon levels. **(D)** Ileum total GLP-1 levels. **(E)** Ileum active GLP-1 levels. The values represent the means ± SEMs. n = 10 per group. All significant p-values were listed and adjusted using the FDR procedure (FDR <0.05).

To identify the effect of liraglutide on GLP-1 secretion, fasting blood samples and ileal samples were collected to determine total and active GLP-1 (7–36,7–37) levels in prediabetic mice (n = 10 per group). As shown in Figures 3A,B,D,E, prediabetic mice presented significantly lower total and active serum and ileal tissue GLP-1 levels than control mice. However, the total and active GLP-1 levels significantly increased after liraglutide treatment, suggesting that GLP-1RAs could improve impaired GLP-1 secretion in prediabetes. Furthermore, there was an obvious increase in serum fasting glucagon levels in prediabetic mice, and liraglutide treatment could significantly decrease the serum glucagon levels (Figure 3C).

**FIGURE 4:**
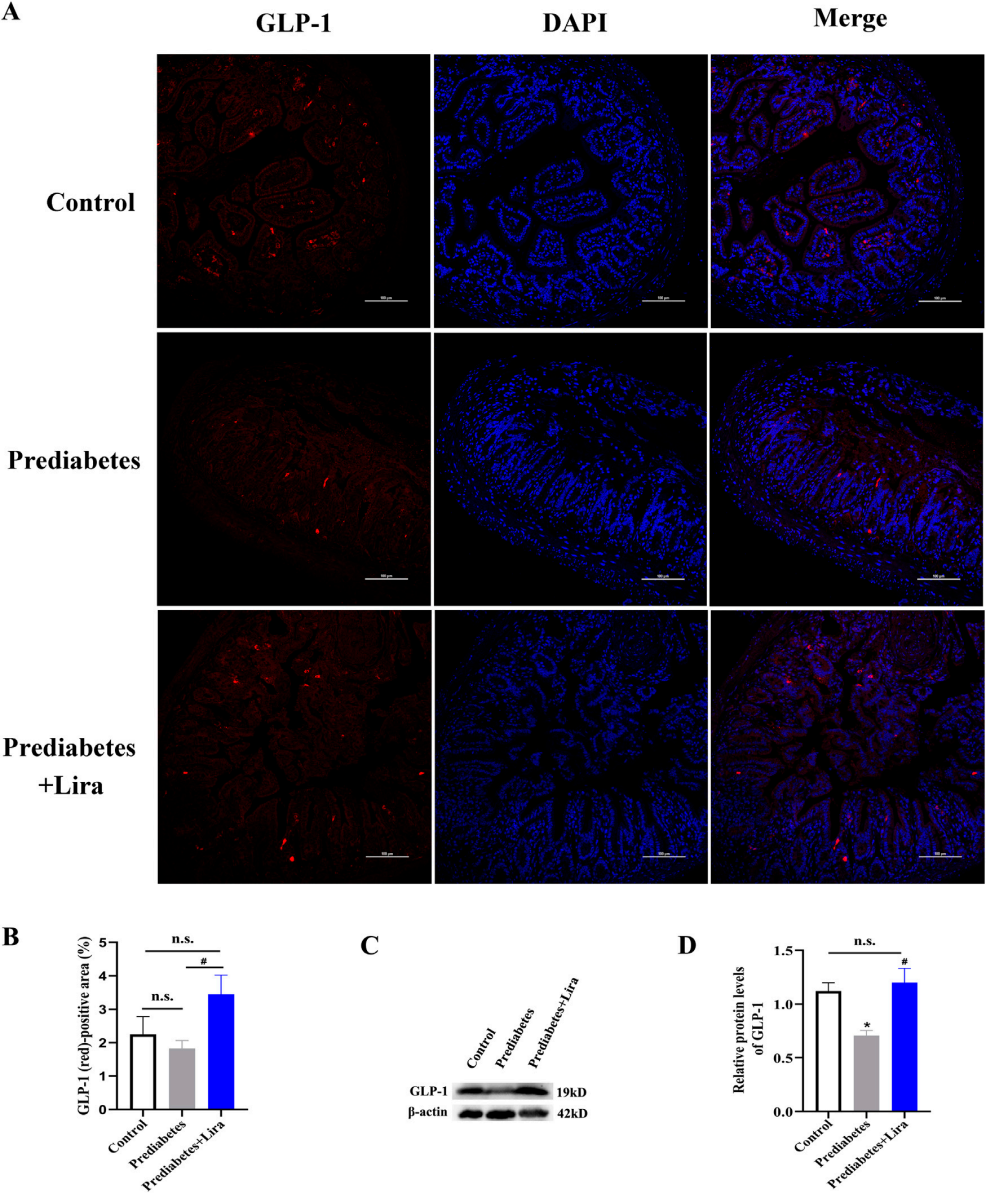
The effect of liraglutide on ileal GLP-1 secretion in prediabetic mice. **(A)** Representative confocal laser scanning microscopy image of the ilea of mice in the three groups. GLP-1 (red), DAPI (blue). **(B)** GLP-1 (red)-positive area (%). Scale bar: 100 µm. **(C)** Representative image of Western blotting results for GLP-1 in the ileum of mice in each of the three groups. β-actin was used as the loading control (n = 10 mice per group). **(D)** Densitometric quantification of GLP-1 bands is shown using ImageJ (version 1.45) with normalization to β-actin. Data are means ± SEM. n = 3-5 per group. All the experiments were performed in duplicate. *P < 0.05 vs. control, ^#^P < 0.05 vs. prediabetes. n.s. denotes no significance.

To further evaluate the effect of liraglutide treatment on GLP-1 secretion, an ileal sample was used to determine GLP-1 secretion by confocal laser scanning microscopy (n = 3–5 per group). Confocal imaging also demonstrated that liraglutide treatment promoted GLP-1 secretion from ileal cells in prediabetic mice (Figures 4A,B). Western blotting analysis confirmed these results (n = 10 per group) (Figures 4C,D). These observations suggest that liraglutide promote GLP-1 secretion through the modulation of ileal cells in prediabetes.

### The effects of liraglutide treatment on gut microbial metabolites in prediabetic mice

**FIGURE 5:**
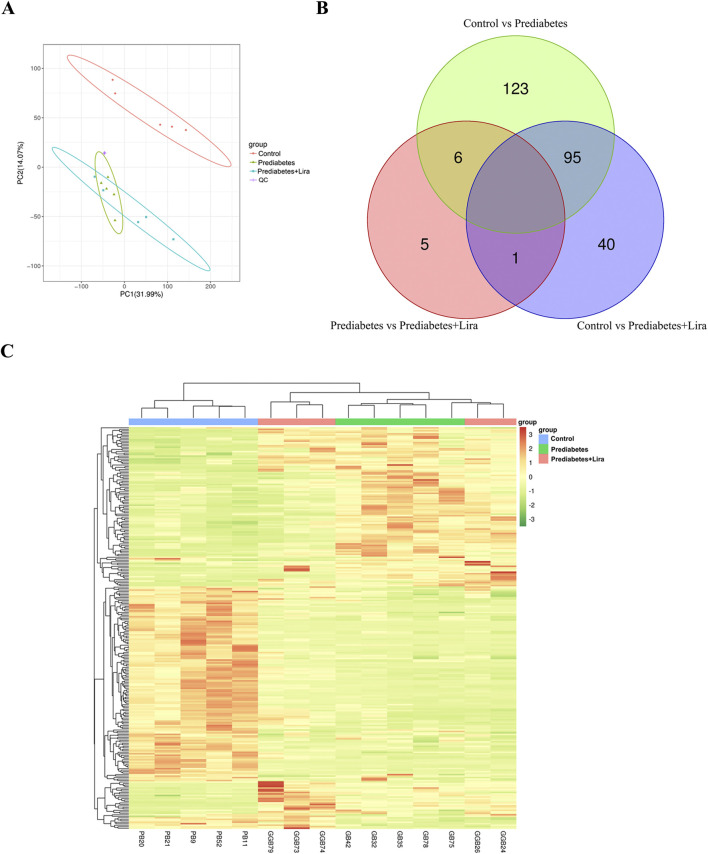
Principal component analysis (PCA), Venn diagram, and heatmap of the ileal contents of the mice in the control, prediabetes, and prediabetes + Lira groups. **(A)** PCA of different groups (n = 10). Each point in the plot represents a sample, and samples from the same group are represented by the same color, while samples from different groups are labeled with different colors. A 95% confidence ellipse is displayed for each group. QC: quality control. **(B)** Venn diagram of differentially abundant metabolites in different groups. Each circle in the figure represents a comparison group. The numbers in the overlapping areas between circles indicate the number of differentially abundant metabolites shared between the comparison groups. The numbers outside the overlapping areas represent the number of unique differentially abundant metabolites for each comparison group. **(C)** Hierarchical clustering heatmap of differentially abundant metabolites in different groups. n = 10 per group.

Gut microbial metabolites have important impacts on pathophysiological processes in human diseases (Krautkramer et al., 2021). To explore the changes in gut microbial metabolites in prediabetic mice and liraglutide-treated prediabetic mice, metabolomic analysis of the ileal contents was performed (n = 5 per group). The PCA score plot (Figure 5A) demonstrates a difference in the distribution of metabolites in the prediabetes or prediabetes + Lira group compared with the control group and that the control mice were clearly separated from the other two groups. There were similar microbial metabolite profiles in the prediabetes and prediabetes + Lira groups. This differential analysis was confirmed via OPLS-DA (Supplementary Figure S1).

A total of 2,104 metabolites were annotated. There were 224 differentially abundant metabolites with a fold change (FC) threshold >1.5 and a VIP value threshold ≥1 (p < 0.05) in prediabetic mice compared with control mice. Among these, the abundances of 96 metabolites were significantly increased, whereas those of 128 metabolites were decreased. Additionally, there were 136 differentially abundant metabolites with significant differences between the prediabetes + Lira group and the control group. Among these, the abundances of 42 metabolites were significantly increased, whereas those of 94 metabolites were decreased. One metabolite with an increased abundance and 11 metabolites with decreased abundances were identified between the prediabetes + Lira and prediabetes groups (Table 1; Figure 5B, Supplementary Material).

**TABLE 1:** Statistics regarding differentially abundant metabolites in different groups.

Group	Total_num	Diff_num	Up_num	Down_num
Control vs. prediabetes	2,104	224	96	128
Control vs. prediabetes + Lira	2,104	136	42	94
Prediabetes vs. prediabetes + Lira	2,104	12	1	11

Group: Grouping information of differentially abundant metabolites; Total_num: Total number of identified metabolites; Diff_num: Number of significantly differentially abundant metabolites; Up_num: Number of metabolites with increased abundance; Down_num: Number of metabolites with decreased abundance.

Only 1 common differentially abundant metabolite (prodigiosin) was decreased in prediabetic + Lira mice compared with prediabetic mice or control mice. The abundances of 6 metabolites, including ethylglyoxalbis (guanylhydrazone), octyl alpha-D-glucopyranoside, PI (14:0/14:1 (9Z)), salbostatin, 6-methylthiopurine 5′-monophosphate ribonucleotide, and maleic acid, were increased in prediabetic mice compared with control mice but decreased in prediabetic + Lira mice (Figure 5B). Striking changes in the metabolic profiles of prediabetic and prediabetic + Lira mice compared with control mice are shown in a cluster heatmap (Figure 5C).

**FIGURE 6:**
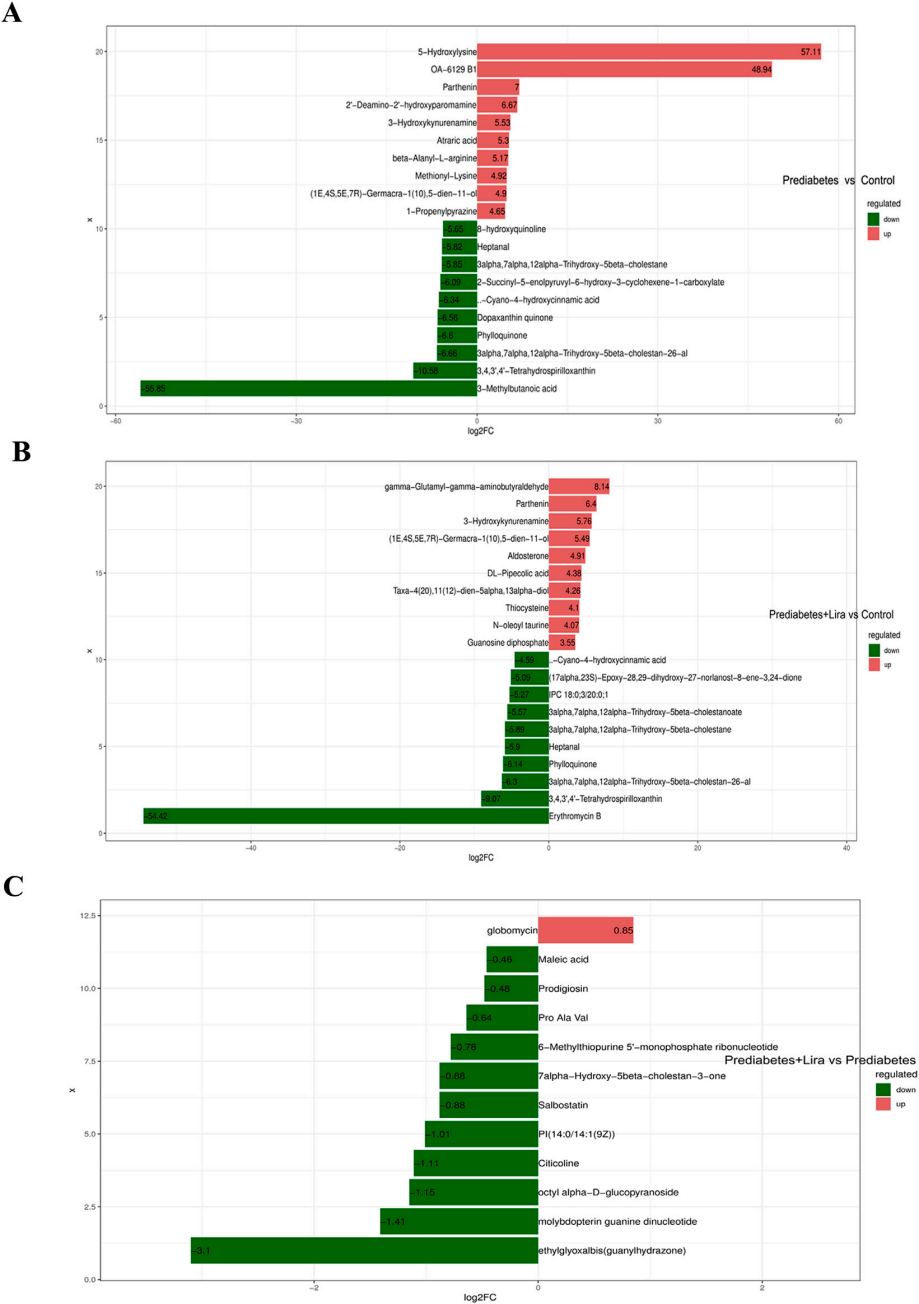
Fold changes (FCs) of differentially abundant metabolites in different groups. The bar plot shows the log-transformed results of the top 10 upregulated and top 10 downregulated metabolites in each group. The labels on each bar represent the names of the metabolites, with upregulated metabolites shown in red and downregulated metabolites in green. The length of each bar represents the log_2_FC value. **(A)** Prediabetes vs. control. **(B)** Prediabetes + Lira vs. control. **(C)** Prediabetes + Lira vs. prediabetes. n = 10 per group.

Analysis of the differentially abundant metabolites (p < 0.05) revealed that the abundances of several metabolites, such as 5-hydroxylysine, OA-6129 B1, parthenin, and atraric acid, were increased and that of 3-methylbutanoic acid was significantly decreased in prediabetic mice compared with control mice (Figure 6A). Compared with control mice, abundances of gamma-glutamyl-gamma-aminobutyraldehyde, parthenin, and 3-hydroxykynurenine were increased, whereas that of erythromycin B was significantly decreased in prediabetes + Lira mice (Figure 6B). The abundance of only 1 distinct metabolite (globomycin) was increased, and those of 11 metabolites, such as ethylglyoxalbis (guanylhydrazone), molybdopterin guanine dinucleotide, octyl alpha-D-glucopyranoside, citicoline, 7alpha-hydroxy-5beta-cholestan-3-one, ProAla Val, and prodigiosin, were decreased in prediabetes + Lira mice compared with prediabetes mice (Figure 6C).

**FIGURE 7:**
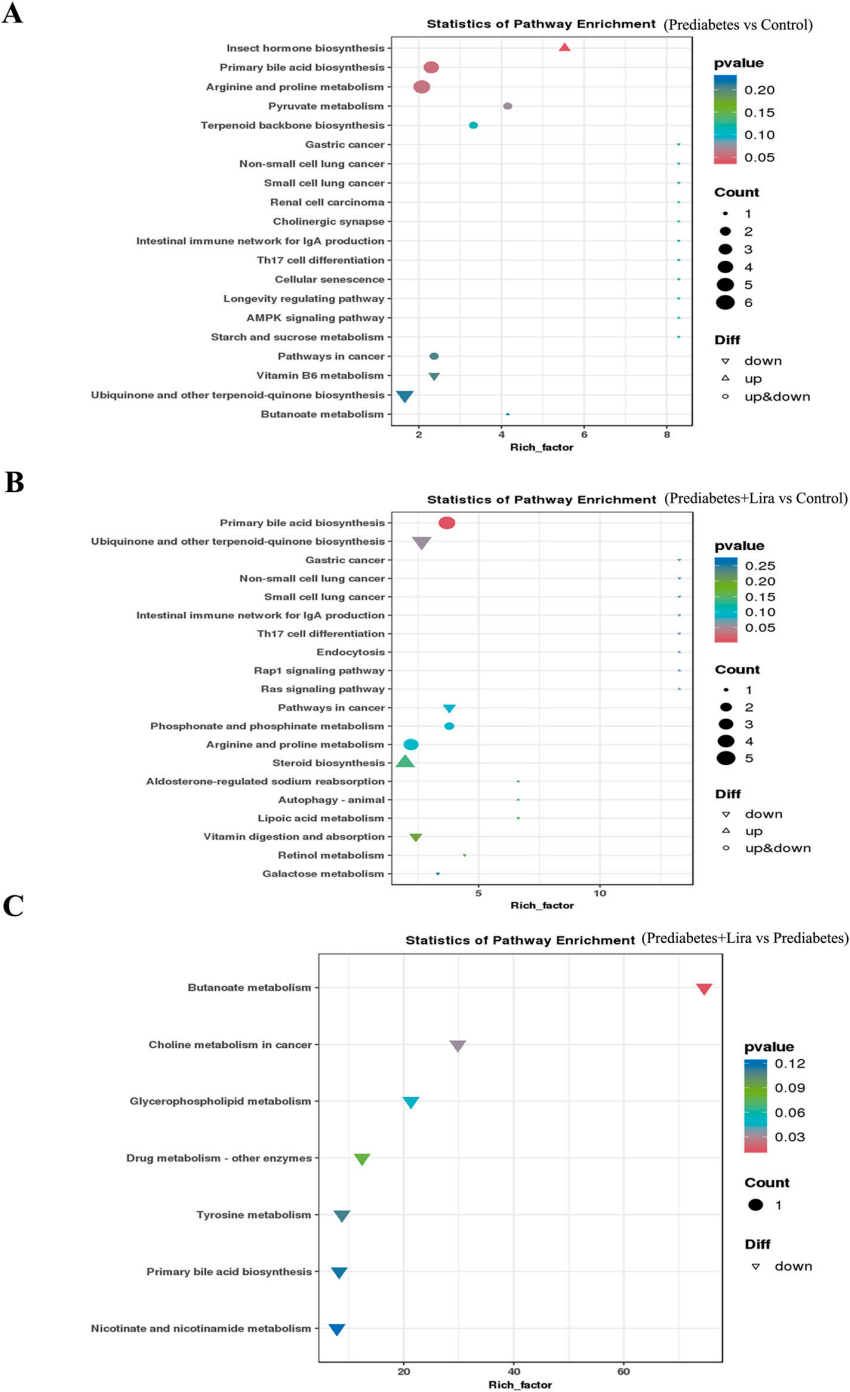
KEGG enrichment plot of differentially abundant metabolites in different groups. Each point in the graph represents a KEGG pathway, with the x-axis representing the enrichment factor (rich factor) and the y-axis representing the pathway names. The color depth of the points represents the P value, with a smaller P value indicating a more significant enrichment of differentially abundant metabolites. **(A)** Prediabetes vs. control. **(B)** Prediabetes + Lira vs. control. **(C)** Prediabetes + Lira vs. prediabetes. n = 10 per group.

The differentially abundant metabolites identified via KEGG pathway enrichment analysis were involved in primary bile acid biosynthesis, arginine and proline metabolism, pyruvate metabolism, and the insect hormone biosynthesis pathway (upregulated) in prediabetic mice compared with control mice (Figure 7A). The primary bile acid biosynthesis and arginine and proline metabolism pathways were also affected in prediabetic + Lira mice compared with control mice (Figure 7B). In addition, the butanoate metabolism pathway was upregulated, and primary bile acid biosynthesis and glycerophospholipid metabolism were downregulated in prediabetic + Lira mice compared with prediabetic mice (Figure 7C). These results indicate that there was a significant change in intestinal microbial metabolites in prediabetic mice and that liraglutide treatment had a strong effect on intestinal microbial metabolites and related signaling pathways.

### Liraglutide treatment promoted gut microbiota modulation in prediabetic mice

**FIGURE 8:**
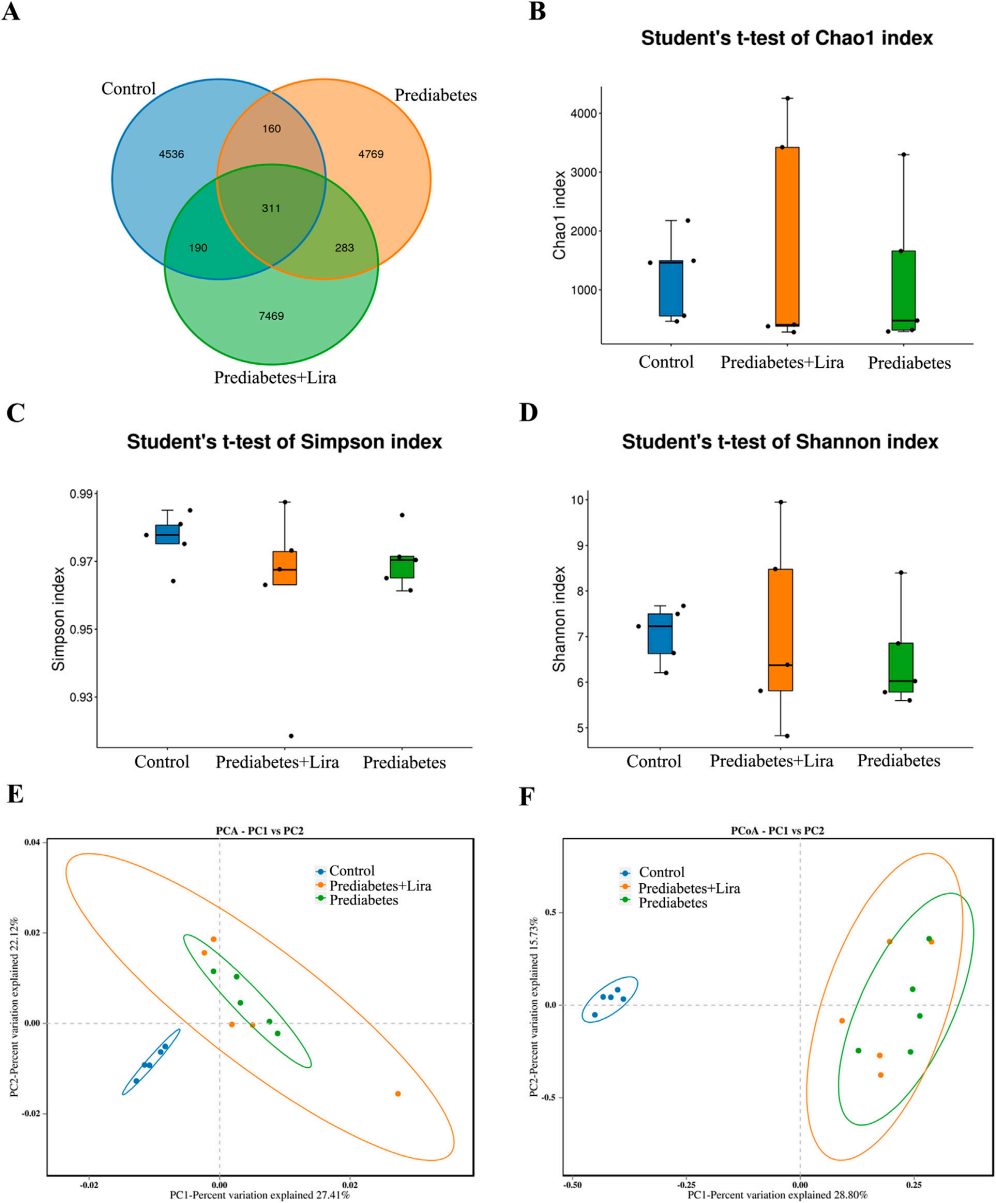
Liraglutide treatment promoted gut microbiota modulation. **(A)** Venn diagram of OUT/ASV numbers in different groups. Each circle represents a group. The overlaps indicate common OTUs/ASVs among groups, and the nonoverlapping area indicates unique OTUs/ASVs in each group. (n = 5/group) **(B)** Alpha diversity analysis: Chao1 index. **(C)** Alpha diversity analysis: Simpson index. **(D)** Alpha diversity analysis: Shannon index. **(E)** Beta diversity analysis: PCA. **(F)** Beta diversity analysis: PCoA. n = 5 per group.

To identify the diversity of the gut microbiota in mice after treatment with liraglutide, microbial diversity analysis of the ileal contents was conducted (n = 5 per group). ASV analysis revealed 4726 and 5052 unique ASVs in the control group and prediabetes group (Figure 8A), respectively. There were 471 common ASVs in the control group and prediabetes group. Additionally, 4929 and 7659 unique ASVs were detected in the mice in the prediabetes group and prediabetes + Lira group, respectively. There were 594 common ASVs in the mice of the prediabetes group and prediabetes + Lira group. A total of 311 common ASVs coexisted in the three groups. These results reveal differential abundances in the intestinal microbiota among the three groups.

Microbial alpha diversity analyses revealed no significant difference of gut bacterial richness in the mice in the prediabetes group and prediabetes + Lira group compared with control group on the basis of the Chao 1 index (Figure 8B). Additionally, the Shannon and Simpson indices show a reduced trend in gut bacterial species diversity and community evenness in the mice in both the prediabetes and prediabetes + Lira groups compared with the control group, but there was no statistic significant difference among three groups (p > 0.05) (Figures 8C,D).

To further assess the species diversity among the different groups, beta diversity analysis was performed. PCA and weighted PCoA revealed substantial compositional differences in the prediabetes and prediabetes + Lira groups compared with the control group. There was a discrete change in the gut microbial community in liraglutide-treated mice compared with prediabetic mice, suggesting a shift in the gut microbial community in prediabetic mice after liraglutide treatment (Figures 8E,F).

**FIGURE 9:**
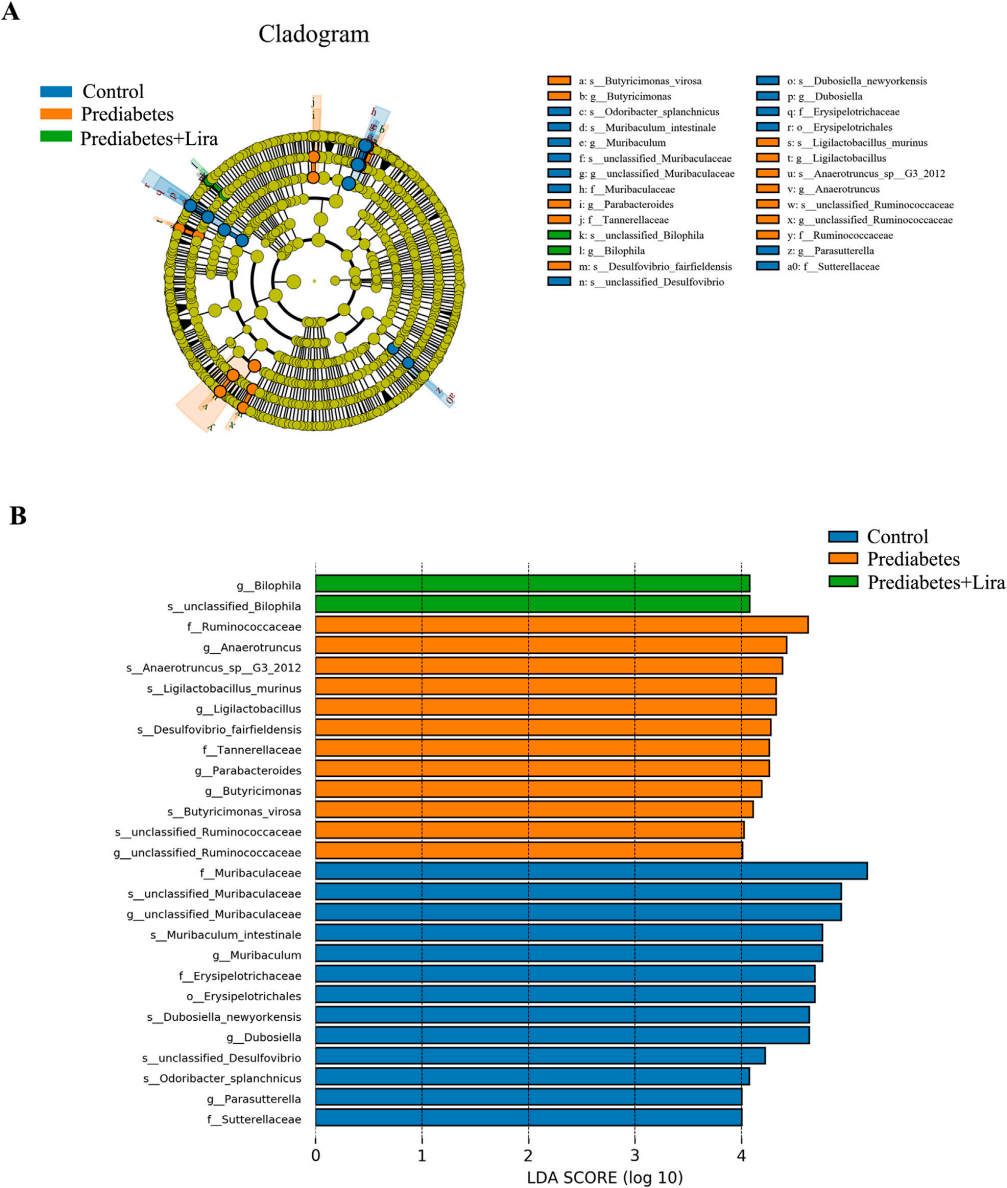
LEfSe analysis between groups. **(A)** LEfSe analysis cladogram diagram. The circles from the center layer to the outward layer represent the taxonomic levels from phylum to species. The size of each dots indicates the relative abundance. Coloring: Species with no significant difference are colored yellow. **(B)** LDA score distribution histogram. n = 5 per group.

LEfSe analysis and Metastats analysis were used to study the significant differences in microbial community abundance between groups at the phylum, class, order, family, genus and species levels (Charpentier et al., 2021). As depicted in Figures 9A,B, Supplementary Figure S2, the LEfSe results revealed significantly different abundances of microbial taxa, as indicated by the LDA score (LDA > 4). The control group presented a significantly greater abundance of gut microbiota, such as *f_Muribaculaceae* (family), *s_Muribaculum_intestinale* (species), *g_Muribaculum* (genus), *f_Erysipelotrichaceae* (family), and *g_Dubosiella* (genus). The prediabetes group presented pronounced increases in the abundances of *f_Ruminococcaceae*, *g_Anaerotruncus*, *s_Anaerotruncus_sp_G3_*2012, *s_Ligilactobacillus_murinus*, *s_Desulfovibrio_fairfieldensis*, *g_Ligilactobacillus*, *g_Parabacteroides*, *g_Butyricimonas*, and *g_unclassified_Ruminococcaceae*. Conversely, the liraglutide treatment group presented greater enrichment of *g_Bilophila* and *s_unclassified_Bilophila*, which are potential biomarkers involved in inflammatory and metabolic diseases (Natividad et al., 2018). To further investigate the differences in microbial community abundance between groups, metastats analysis was performed (White et al., 2009). Metastats analysis indicated a greater abundance of *Unclassified_Clostridia_UCG_014*, *Eisenbergiella*, and *Dorea* at the genus level after liraglutide treatment, whereas the abundances of *Anaerotruncus*, *unclassified_Clostridia_vadinBB60_group*, *Lachnospiraceae_UCG-001*, and *Parabacteroides* significantly decreased (Supplementary Figure S3). These alterations in microbial abundance suggest that liraglutide treatment has an important effect on the gut microbiota composition in prediabetic mice.

**FIGURE 10:**
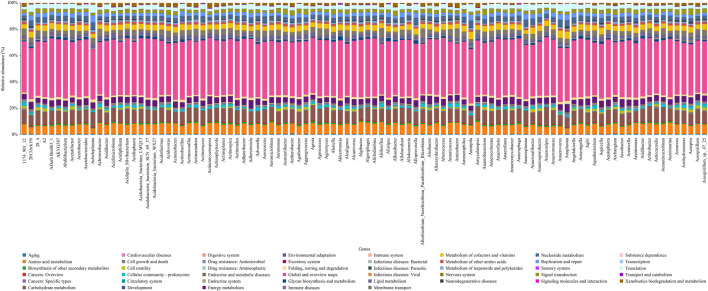
Differentially active metabolic pathways based on KEGG functional prediction at the genus level.

To assess the effect of the differentially abundant gut microbiota on metabolic function, KEGG-based functional pathway analysis was performed using PICRUSt2 software. The KEGG analysis revealed that the differentially abundant gut microbiota was involved mainly in amino acid metabolism, carbohydrate metabolism, metabolism of cofactors and vitamins, and energy metabolism signaling pathways (Figure 10).

## Discussion

Our results demonstrate that liraglutide decreases body weight and dysglycemia, increases insulin and GLP-1 secretion, and inhibits inflammation in prediabetic mice. liraglutide improved metabolic parameters such as body weight, dysglycemia, and insulin resistance, which was consistent with previous study (Charpentier et al., 2021). The present study showed that prediabetic KKay mice had significantly decreased ileal and serum GLP-1 levels, while ileal and serum GLP-1 levels were increased after liraglutide treatment. The stimulatory effect was also found in GLUTag cells (*in vitro*) or intestinal L cells in type 2 diabetic rats and human (*in vivo*) with exendin-4 or liraglutide treatment (Kappe et al., 2013; Chun-hua et al., 2013; Kramer et al., 2017). It has been reported that the GLP-1 receptor is expressed in the jejunum, ileum, and colon, and that administration of the GLP-1 receptor agonist (exendin-4) to mice can activate the GLP-1 receptor and induce early gene c-fos expression (Kedees et al., 2013). These findings indicate that GLP-1RA liraglutide has also a protective effect on impaired GLP-1 secretion to some degree in prediabetes. However, the underlying mechanism by which liraglutide enhances GLP-1 secretion in the ileum warrants further investigation.

A growing body of evidence has shown that chronic low-grade inflammation plays a critical role in prediabetes and diabetes (Saukkonen et al., 2018; Weaver et al., 2021). In consistent with previous results, the KKay prediabetic mice exhibit significant increases in serum and ileum levels of inflammatory factors such as IL-1β, IL-6, LPS, NF-κB, and TNF-α, while liraglutide can reduce these inflammatory factors, suggesting an inhibitory effect of liraglutide on the body’s inflammatory response in prediabetic state. The underlying mechanism may be involved in the anti-inflammatory effect of liraglutide on the intestinal immune system, such as TReg cells and Th1 lymphocytes (Charpentier et al., 2021).Additionally, there was an increased fasting serum glucagon in prediabetic mice, which glucagon played a pathophysiologic role in metabolic disease such as diabetes and obesity (Knop et al., 2012; Morita et al., 2021). liraglutide treatment decreased the serum glucagon levels in prediabetic mice in line with previous study (Jorsal et al., 2016; Mashayekhi et al., 2024).

The gut microbiota and its metabolites play critical roles in regulating host metabolism, immune function, insulin resistance and overall health (Takeuchi et al., 2023; Krautkramer et al., 2021). An increasing number of studies has shown gut microbial dysbiosis and altered abundances of microbial metabolites, such as butyrate and butyrate-producing bacteria, in individuals with prediabetes (Allin et al., 2018; Gravdal et al., 2023; Wu et al., 2020).

We observed that liraglutide treatment led to 224 differentially abundant metabolites in prediabetic mice compared with control mice, 96 metabolites of which showed significantly increased abundances, whereas those of 128 metabolites were decreased. Analysis of the differentially abundant metabolites revealed that globomycin abundance was increased in prediabetes, and that of 11 metabolites, such as ethylglyoxalbis (guanylhydrazone), molybdopterin guanine dinucleotide, octyl alpha-D-glucopyranoside, citicoline, 7alpha-hydroxy-5beta-cholestan-3-one, ProAla Val, and prodigiosin, was decreased after treatment with liraglutide.

Globomycin is a natural antibiotic, and the effect of liraglutide on globomycin suggests that liraglutide has antimicrobial properties, thereby presumably modulating inflammatory and metabolic processes (Olatunji et al., 2020; Oves-Costales et al., 2023). Ethylglyoxalbis (guanylhydrazone), a chemical substance, is an inhibitor of S-adenosylmethionine decarboxylase, a critical enzyme in the polyamine biosynthesis (Seppanen et al., 1984). Polyamines were produced by intestinal bacteria and implicated in numerous pathophysiological processes including cancer and lifespan (Chia et al., 2022). Prior study reported that there was a declined polyamine level with age, and supplement of polyamine could have a beneficial effect on cardioprotection and lifespan (Mashayekhi et al., 2024). However, another study revealed that increased polyamines promoted tumor growth and progression, and inhibition of polyamine could serve as a therapeutic strategy for renal cancer (Ochocki et al., 2018). Our findings showed a reduction in ethylglyoxalbis following liraglutide treatment. Further research will investigate the significance of liraglutide on ethylglyoxalbis and its role in polyamine production. These differentially abundant metabolites are involved mainly in primary bile acid biosynthesis, arginine and proline metabolism, pyruvate metabolism, and the insect hormone biosynthesis pathway. For example, 7alpha-hydroxy-5beta-cholestan-3-one is an intermediate product of primary bile acid synthesis (Offei et al., 2019). Our findings show that liraglutide activates the butanoate metabolism pathway. Previous studies have demonstrated that bacterial butyrate production and the number of butyrate-producing bacteria are decreased in individuals with prediabetes and that supplementation with butyrate can improve insulin sensitivity (Wu et al., 2020; Gao et al., 2009; Gravdal et al., 2023). These findings indicate that GLP-1RAs influence the production of these intestinal microbial metabolites and their related signaling pathways.

To further investigate the effect of liraglutide on the gut microbiota composition in prediabetic mice, we focused on the changes in the gut microbiota between prediabetic and liraglutide-treated prediabetic mice. LEfSe analysis with an LDA score (LDA > 4) revealed an imbalance in microbial diversity and abundance in prediabetic mice, such as increased abundance of *f_Ruminococcaceae*, *g_Anaerotruncus, s_Anaerotruncus_sp_G3_*2012, *s_Ligilactobacillus_murinus, s_Desulfovibrio_fairfieldensis, g_Ligilactobacillus, g_Parabacteroides, g_Butyricimonas,* and *g_unclassified_Ruminococcaceae*.

We observed an increase in *f_Ruminococcaceae* abundance in mice with prediabetes, which is consistent with the results of earlier clinical studies (Pinna et al., 2021; Allin et al., 2018). Studies have indicated an inverse association between *Ruminococcaceae* abundance and glucose metabolism (Allin et al., 2018). A population study confirmed an inverse association between the butyrate-producing family *Ruminococcaceae* and insulin resistance (Chen et al., 2021). The abundance of *Ruminococcaceae* was significantly higher in patients with Type 1 diabetes and showed a negative correlation with hemoglobin A1c levels (Abuqwider et al., 2023), which is not consistent with other study (Huang et al., 2018). This differential finding may be due to confounding factors such as race, age, sex, medication (Santos-Marcos et al., 2023). Further investigation is necessary to elucidate the underlying mechanisms by which *Ruminococcaceae* influences glucose metabolism.

A recent study reported a high abundance of the genus *Anaerotruncus* in obese males with insulin resistance (Sen et al., 2024). Nevertheless, the interactive mechanism of these bacteria and glucose metabolism was not elucidated, and further studies are required to thoroughly examine how these bacteria affect the pathophysiological process of prediabetes.

Liraglutide treatment resulted in a greater enrichment of *g_Bilophila* and *s_unclassified_Bilophila* in the gut microbial communities in prediabetic mice. *g_Bilophila,* a potential pathogenic bacterium, is involved in the process of bile acid dysmetabolism, such as primary biliary cholangitis and cholangiolithiasis (Tang et al., 2018; Liang et al., 2016). The potential mechanism was associated with taurine metabolism (Liang et al., 2016). While, liraglutide treatment could increase the high risk of clinical cholelithiasis (Gameil et al., 2024; Moll et al., 2024). We speculated that the greater enrichment of *Bilophila* may be play a critical role in liraglutide-related cholelithiasis.

Metastats analysis indicated that liraglutide treatment significantly increased the abundance of bacteria such as *unclassified_Clostridia_UCG_014*, *Eisenbergiella*, and *Dorea* at the genus level. *Eisenbergiella* is a genus of Firmicutes in the class Clostridia and is more abundant in older people, suggesting an association between longevity and *Eisenbergiella* abundance (Chen et al., 2024). A previous study indicated that the abundance of *Dorea* was increased in individuals with prediabetes and that *Dorea* abundance was positively correlated with fasting plasma glucose levels. (Allin et al., 2018; Fei et al., 2019). Another study revealed that *Dorea* abundance was high in the Danish population but not in Indian individuals with prediabetes, suggesting an ethnicity difference (Pinna et al., 2021). However, the specific effects of liraglutide on *Dorea* need further study.

Additionally, liraglutide decreased the gut bacterial abundance of *unclassified_Clostridia_vadinBB60_group*, *Lachnospiraceae_UCG-001*, *Anaerotruncus*, and *Parabacteroides* in prediabetic mice, and these bacteria are abundant in the prediabetic population (Pinna et al., 2021; Chang et al., 2024). *Lachnospiraceae* is a pathogenic bacterium whose abundance is increased in diabetic KKay mice, and angelica polysaccharide (AP) treatment improved hyperglycemia and decreased *Lachnospiraceae* abundance (Tang et al., 2023). *Anaerotruncus* abundance was negatively correlated with butyric acid and propionic acid levels in piglets, suggesting a complicated interaction between *Anaerotruncus* and butyric/propionic-producing bacteria (Liu et al., 2018). The effect of liraglutide provides a potential therapeutic target for treating prediabetes through the modulation of specific gut microbiota. Recently, extensive research has demonstrated that remodeling the gut microbiota has emerged as a therapeutic strategy for reversing dysglycemia, including the use of probiotics and fecal microbiota transplantation (Yang et al., 2023; Zhang et al., 2024a).

Liraglutide treatment induced differential gut microbiota compositions and related metabolic pathways in mice with prediabetes. These pathways are involved in amino acid metabolism, carbohydrate metabolism, the metabolism of cofactors and vitamins, and energy metabolism signaling pathways. Recent research has indicated that the gut microbiome potentially influences intestinal carbohydrate metabolism and improves insulin resistance, suggesting that intestinal carbohydrate metabolism plays an important role in insulin sensitivity (Takeuchi et al., 2023). Hence, these findings elucidate the potential of liraglutide to ameliorate prediabetes progression and insulin resistance through the modulation of the gut microbiota.

Eventually, these results enhance our understanding that liraglutide influences glucose metabolism, body weight, GLP-1 secretion, gut microbiota in prediabetes. However, several questions remain unanswered. Owing to the lack of weight-matched or pair-fed controls, the observed changes in the gut microbiota/metabolites could potentially be driven by the weight loss induced by liraglutide, rather than representing a direct effect of the drug itself. There is a complex interaction between gut microbiota and body weight; some bacteria, such as *Akkermansia*

*muciniphila*, or fecal microbiota transplantation promote weight loss, while others do not (Everard et al., 2013; Van Hul and Cani, 2023). Future investigations are needed to ascertain the role of gut microbiota in the effects of liraglutide on prediabetes.

## Conclusion

The present study demonstrates that the GLP-1RA liraglutide exerts beneficial effects by improving dysglycemia and body weight, inhibiting inflammation, and modulating gut microbiota in prediabetic mice, potentially contributing to delaying or preventing the progression from prediabetes to overt diabetes. Therefore, the potential interaction mechanism between the gut microbiota and liraglutide deserves further investigation.

## Data Availability

The study raw data of the 16S rDNA sequences are deposited in the China National Center for Bioinformation, accession number PRJCA051399; available at https://ngdc.cncb.ac.cn/search/all?q=PRJCA051399.
