# Supplementary material for: Effect of liraglutide on the dysglycemia, inflammation, and gut microbiota in prediabetic KKay mice
Source: Front Pharmacol. 2025 Nov 28;16:1714859. doi: 10.3389/fphar.2025.1714859 (PMC12698571; doi:10.3389/fphar.2025.1714859)

**Supplementary information for**

**Effect of** **liraglutide on the** **dysglycemia, inflammation, and gut microbiota in prediabetic KKay mice**

Ying Zhang^1^, Xiaoxiao Yang^1^, Ping Yang^1^, Huihuan Sun^1^, Lijuan Chen^1^, Xiaojun Zhang^2^, Shudong Liu^1^**^*^**

**^*^**Correspondence author: lsd008@126.com

**Supplementary Figure legends**

**Figure S1 OPLS-DA score plot for each group. (**A) Prediabetes vs. control. (B) Prediabetes + Lira vs. control. (C) Prediabetes + Lira vs. prediabetes. The x-axis (t1) represents the predictive component (between-group differential component), the y-axis (to1) represents the orthogonal component (within-group differential component), and the horizontal y-axis percentage represents the proportion of variance explained by each component. The model parameters, including R2X, R2Y, Q2Y, RMSEE (root mean square error of estimation), pre (number of predictive components), and ort (number of orthogonal components), are annotated below the graph. (D) A permutation test using to assess the reliability of the OPLS-DA model. Two groups (prediabetes + Lira and prediabetes) are randomly shuffled (permuted), and the OPLS-DA model is built using the permuted groups. The R2Y and Q2Y values are calculated for each permutation.

**Figure S2 The relative abundance of Bilophila in each group (n =5 mice per group)**. Boxplot displayed the minima and maxima, median (center line), and 25% and 75% quantiles (box). Statistical significance was assessed by Kruskal-Wallis test then corrected as false discovery rate (FDR) using the Benjamin-Hochberg method. Asterisk denotes significant p value (FDR-corrected, p < 0.05). n.s., no significance.

**Figure S3**. **Metastats analysis of microbial community abundance between groups at the genus level.** (A) Control vs. prediabetes. (B) Prediabetes vs. prediabetes + lira (n = 5/group).

Figure S1


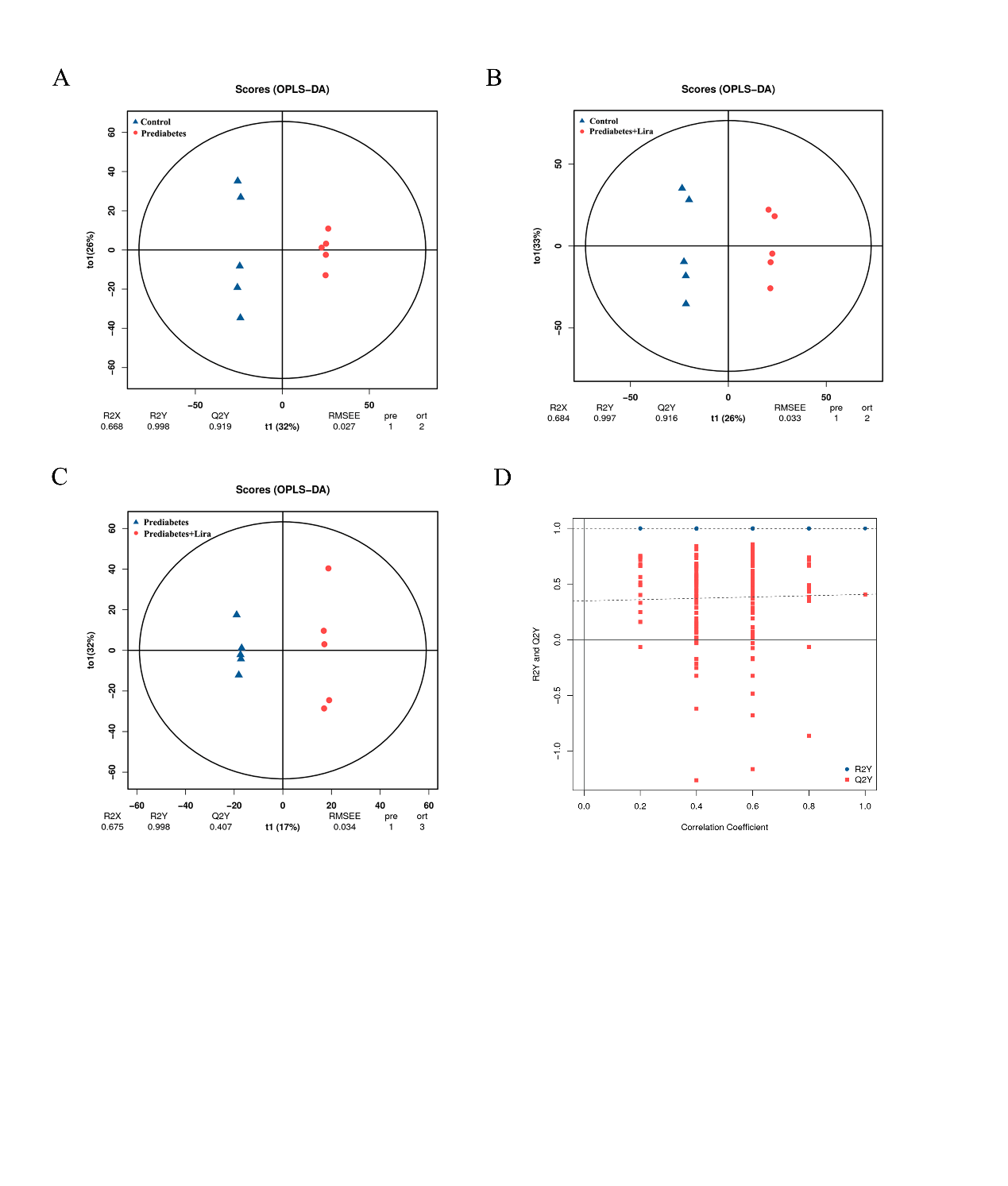


Figure S2


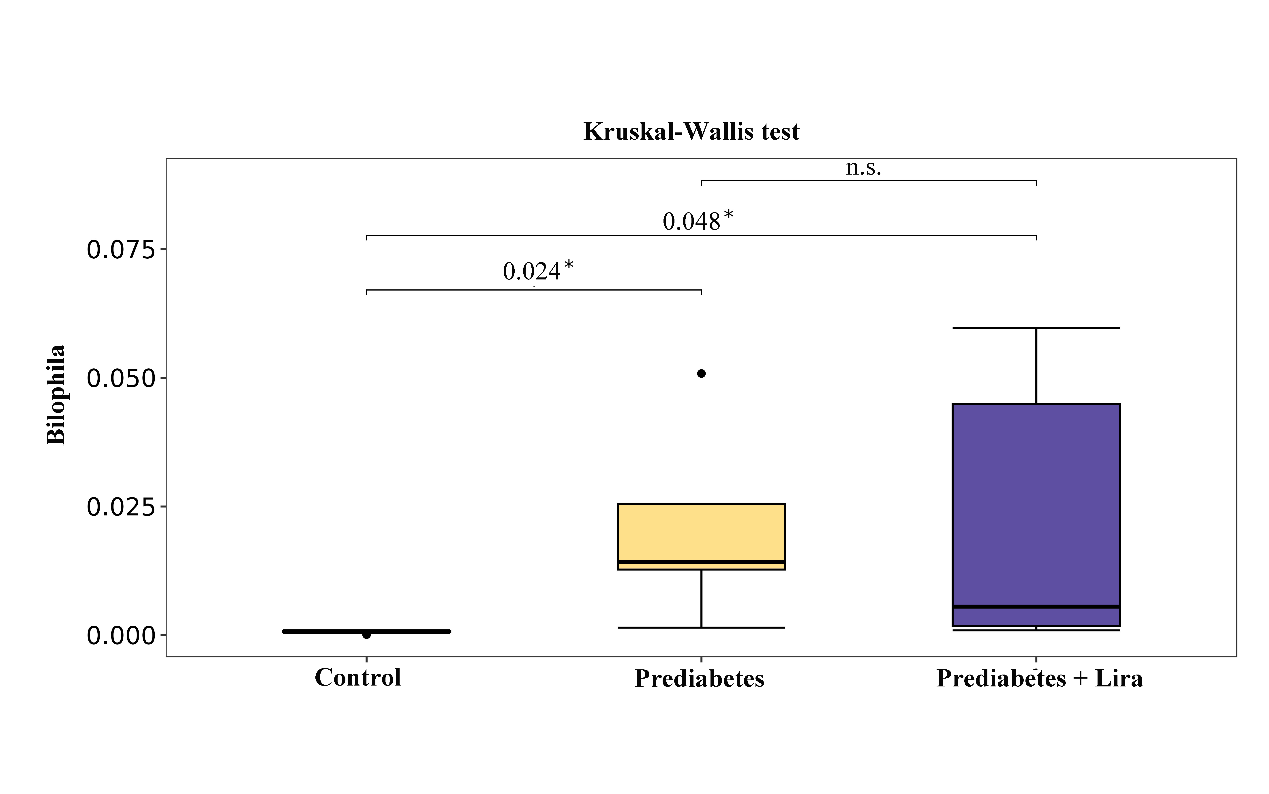


Figure S3


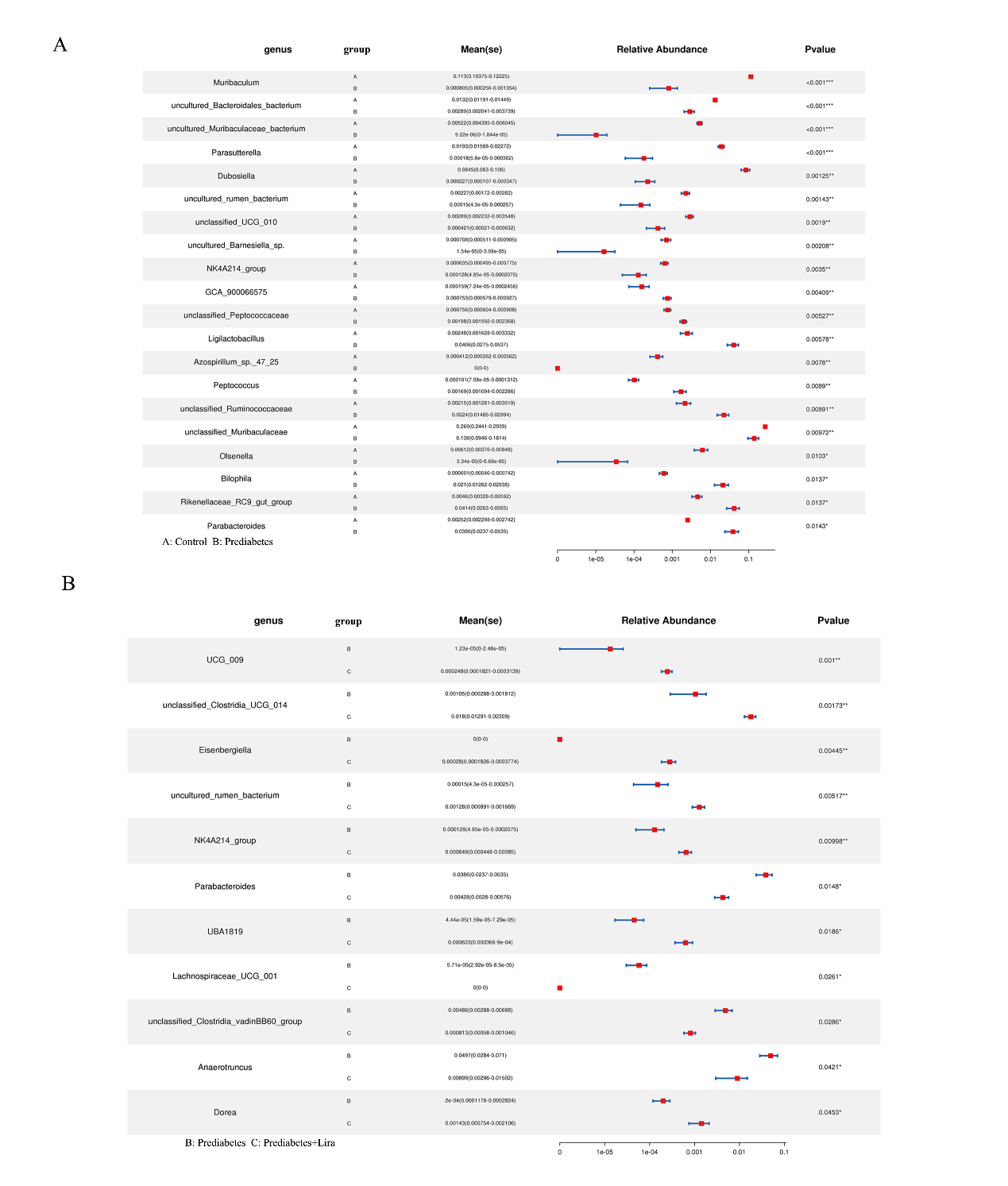

Supplement: Supplementary file 2 [file DataSheet1.docx]
